# Monosodium urate crystal induced macrophage inflammation is attenuated by chondroitin sulphate: pre-clinical model for gout prophylaxis?

**DOI:** 10.1186/1471-2474-15-318

**Published:** 2014-09-27

**Authors:** Eric W Orlowsky, Thomas V Stabler, Eulàlia Montell, Josep Vergés, Virginia Byers Kraus

**Affiliations:** Department of Medicine, Division of Rheumatology, Duke University School of Medicine, Durham, NC USA; Duke Molecular Physiology Institute, Duke University School of Medicine, Durham, NC USA; Pre-Clinical R&D Area, Pharmascience Division, Bioibérica, Barcelona Spain

## Abstract

**Background:**

Chondroitin Sulphate (CS), a natural glycosaminoglycan of the extracellular matrix, has clinical benefit in symptomatic osteoarthritis but has never been tested in gout. *In vitro*, CS has anti-inflammatory and positive effects on osteoarthritic chondrocytes, synoviocytes and subchondral bone osteoblasts, but its effect on macrophages is unknown. The purpose of our study was to evaluate the *in vitro* effects of CS on monosodium urate (MSU)-stimulated cytokine production by macrophages.

**Methods:**

THP-1 monocytes were differentiated into mature macrophages using a phorbol ester, pretreated for 4 hours with CS in a physiologically achievable range of concentrations (10–200 μg/ml) followed by MSU crystal stimulation for 24 hours. Cell culture media were analyzed by immunoassay for factors known to be upregulated during gouty inflammation including IL-1β, IL-8 and TNFα. The specificity of inflammasome activation by MSU crystals was tested with a caspase-1 inhibitor (0.01 μM-10 μM).

**Results:**

MSU crystals ≥10 mg/dl increased macrophage production of IL-1β, IL-8 and TNFα a mean 7-, 3- and 4-fold respectively. Induction of IL-1β by MSU was fully inhibited by a caspase-1 inhibitor confirming inflammasome activation as the mechanism for generating this cytokine. In a dose-dependent manner, CS significantly inhibited IL-1β (p = 0.003), and TNFα (p = 0.02) production from macrophages in response to MSU. A similar trend was observed for IL-8 but was not statistically significant (p = 0.41).

**Conclusions:**

CS attenuated MSU crystal induced macrophage inflammation, suggesting a possible role for CS in gout prophylaxis.

**Electronic supplementary material:**

The online version of this article (doi:10.1186/1471-2474-15-318) contains supplementary material, which is available to authorized users.

## Background

Gout is the most common cause of arthritis in men after osteoarthritis. Its prevalence is on the rise and thought to affect around 4% of the total US population[1]. Patients have fewer flares when their serum uric acid is maintained below 6.0 mg/dl[2]. However, initiation of urate-lowering therapy can often lead to an increase in the frequency and severity of flares[3, 4]. Consequently, both the European League Against Rheumatism (EULAR) and the American College of Rheumatology (ACR) have recommended the use of prophylactic agents when initiating urate-lowering therapy[5, 6]. Oral colchicine, low-dose non-steroidal anti-inflammatory drugs (NSAIDs) and daily corticosteroids have all been recommended[7], but all are associated with intolerances or adverse effects[4]. Thus, the identification of new agents for treating or preventing gout flares would be of great clinical value.

Gouty inflammation is initiated when monosodium urate (MSU) crystals are taken up by macrophages or other cells in the joints[8]. This results in assembly of the NLRP3 inflammasome, a multimeric protein complex responsible for activating caspase-1, which in turn cleaves pro-IL-1β leading to production and secretion of active IL-1β[9]. Other factors are upregulated during gouty inflammation, including IL-8 and TNFα[8].

Chondroitin Sulphate (CS), a natural glycosaminoglycan of the cartilage extracellular matrix[10], is of clinical benefit in symptomatic osteoarthritis[11] but results are mixed[12, 13]. The effects of CS have never been tested in gout. *In vitro*, CS has anti-inflammatory and positive effects on osteoarthritic chondrocytes, synoviocytes and subchondral bone osteoblasts[14], but its effect on macrophages is unknown. On the other hand, *in vivo*, CS given orally prevents hepatic NF-κB nuclear translocation, suggesting that systemic CS may elicit an anti-inflammatory effect in many tissues besides the joint[14]. There is preliminary evidence in human beings that CS may be of benefit in other diseases where inflammation is an essential component such as psoriasis and atherosclerosis[14]. The purpose of our study was to evaluate the *in vitro* effects of CS on MSU-stimulated cytokine production by macrophages.

## Methods

### Cell culture

We established an *in vitro* cell culture system using the human monocytic cell line, THP-1 (ATCC TIB-202), grown in RPMI 1640 with HEPES and supplemented with glucose, pyruvate, 2-mercaptoethanol, 10% FBS and penicillin/streptomycin as recommended by ATCC. These cells were grown to a density of 1.5 × 10^6^ cells/ml in a 75 cm flask and then were induced to differentiate into mature macrophages using 12-O-tetradecanoylphorbol-13-acetate (Enzo Life Sciences) at a concentration of 0.5 μM for 3 hours[15]. Following induction, cells were washed with PBS and then plated into 12-well tissue culture plates at a density of 6 x10^5^ cells/well and incubated overnight in normal media. Prior to any activation studies, cells were washed with PBS followed by the addition of 0.5 ml of serum free Opti-MEM per well.

### Macrophage activation studies

Different concentrations of Monosodium Urate (MSU) crystals (Enzo Life Sciences) in a physiological range (concentrations of serum uric acid that are possible in humans, i.e. up to 20 mg/dl)[16, 17] were initially tested to establish conditions for inducing pro-inflammatory cytokines from activated macrophages. MSU crystals (2.5 to 20 mg/dl) were added to the differentiated cells grown in Opti-MEM and incubated for 24 hours in 10% CO2. Cell culture media were then removed and stored at −80°C until analyzed by immunoassay for IL-1β (high-sensitivity assay R&D Systems), and TNFα and IL-8 (run as part of a human proinflammatory 9-plex by Meso Scale Discovery, MSD). All samples yielded measurable concentrations; 23 of 46 values for IL-8 were out of range high but could be readily extrapolated as they were within the linear range of the assay. The intra and inter-assay coefficients of variation (CV) for IL-1β were 2.85% and 4.87% respectively as reported by the manufacturer. However, no CVs were reported by the manufacturer for the MSD kit. The intra-assay CVs were 4.85% for TNFα, and 2.77% for IL-8 according to our calculations.

In order to identify the component of IL-1β production attributable to inflammasome activation, a commercially available cell-permeable caspase-1 inhibitor (EMD Millipore catalog#400011, sequence: Ac-AAVALLPAVLLALLAPYVAD-CHO) was used. Cells were pre-treated with various concentrations of the inhibitor (0.01-10 μM) for six hours prior to stimulation with MSU crystals (20 mg/dl). This high concentration of MSU was tested to provide a stringent test of caspase inhibition. After stimulation of macrophages for 24 hours as described above, the cell culture media were analyzed for IL-1β.

### CS inhibition studies

To test for anti-inflammatory effects of CS, macrophages were pretreated with highly purified bovine chondroitins 4 and 6 sulfate of ≥98% purity, and with an average molecular weight of ~ 15–16 kDa (Bioibérica, Barcelona, Spain) for 4 hours prior to the addition of MSU crystals (10 mg/dl). A range of doses of CS (10–200 μg/ml) that approximate physiological conditions[18, 19] were tested. Culture media were collected at 24 hours and IL-1β, IL-8 and TNFα concentrations were analyzed as above. The IL-1β data represent the aggregate of 7 total replicates over 4 independent experiments; the IL-8 and TNFα data represent 4 total replicates from 4 separate experiments.

### Endotoxin assay

To test for the presence of endotoxin in the experimental reagents utilized for these experiments, we used Pyrogene Recombinant Factor C Endotoxin Assay (Lonza) according to the manufacturer's instructions. This assay utilizes a recombinant Factor C, which when activated by endotoxin binding reacts with a fluorogenic substrate to produce a fluorescent signal in direct proportion to the amount of endotoxin in the sample.

### Statistical analysis

Fold activation of cytokines was determined comparing the negative controls (no added MSU) to MSU with results expressed as mean % control. CS effects on MSU induced cytokine concentrations were expressed as a mean percent of the MSU only condition (set to 100%). Statistical significance was determined by one-way ANOVA with Dunnett’s post-hoc test. Linear trend analyses of these data were performed to assess for a CS dose response. Analyses were performed using GraphPad Prism software (San Diego, CA). Linear trend analyses were performed using JMP 9 (SAS). Results were considered significant for p < 0.05.

## Results and discussion

**Figure 1:**
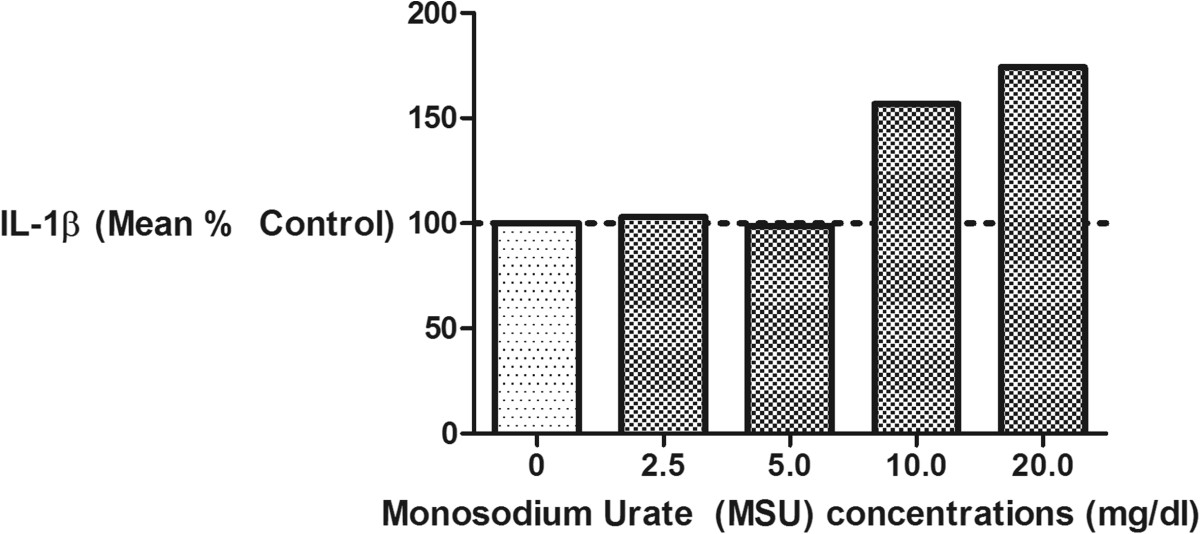
**Exposure of macrophages to high physiological concentrations of monosodium urate (MSU) crystals stimulated IL-1β production.** THP-1 macrophages were exposed to MSU crystals of varying concentrations (range 0–20 mg/dl) for 24 hours. MSU concentrations of ≥ 10 mg/dl consistently induced IL-1β production as shown here in this representative experiment; this prompted us to choose 10–20 mg/dl for all subsequent experiments. Results are expressed as a percent of the negative control (left bar with no MSU).

**Figure 2:**
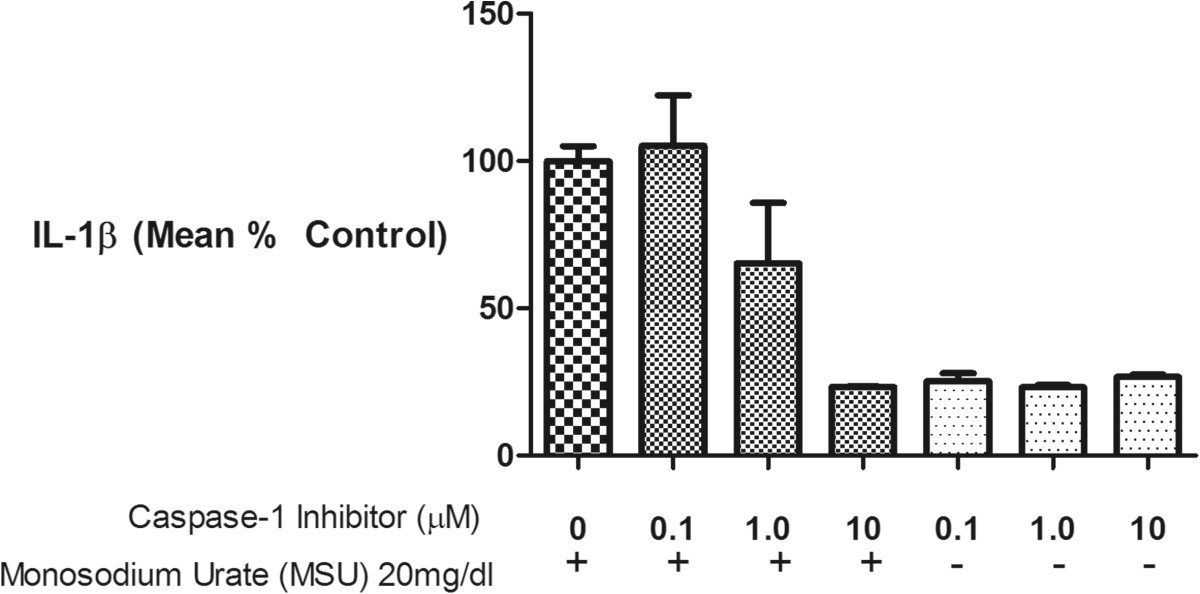
**The origin of IL-1β from MSU stimulated macrophages was consistent with NLRP3 inflammasome activation.** THP-1 macrophages were pretreated for 6 hours with varying concentrations of a Caspase-1 inhibitor (range 0–10 μM) followed by stimulation with MSU (20 mg/dl) for 24 hours. In response to the Caspase-1 inhibitor, IL-1β was reduced in a dose-deoendent manner to the level produced by THP-1 in the absence of MSU (far right three bars). Results are expressed as a percent of the positive control—MSU in the absence of Caspase-1 (first bar on left).

All cell culture reagents, including the MSU and CS, were tested for the presence of endotoxin by our laboratory or the manufacturer, and all were found to contain less than 0.03 EU/ml endotoxin. Increasing concentrations of MSU crystals led to increasing IL-1β production (Figure 1). Specifically, MSU concentrations of 10 mg/dl and greater increased IL-1β production by macrophages; thus, concentrations of 10–20 mg/dl were used for subsequent experiments.To stringently assess the mechanism of IL-1β production in macrophages in response to MSU, cells were stimulated with a high concentration of MSU, 20 mg/dl, with pre-incubation with varying concentrations of a caspase-1 inhibitor. IL-1β production was fully inhibitable by the caspase-1 inhibitor in a dose dependent manner confirming inflammasome activation as the source of this cytokine (Figure 2).

**Figure 3:**
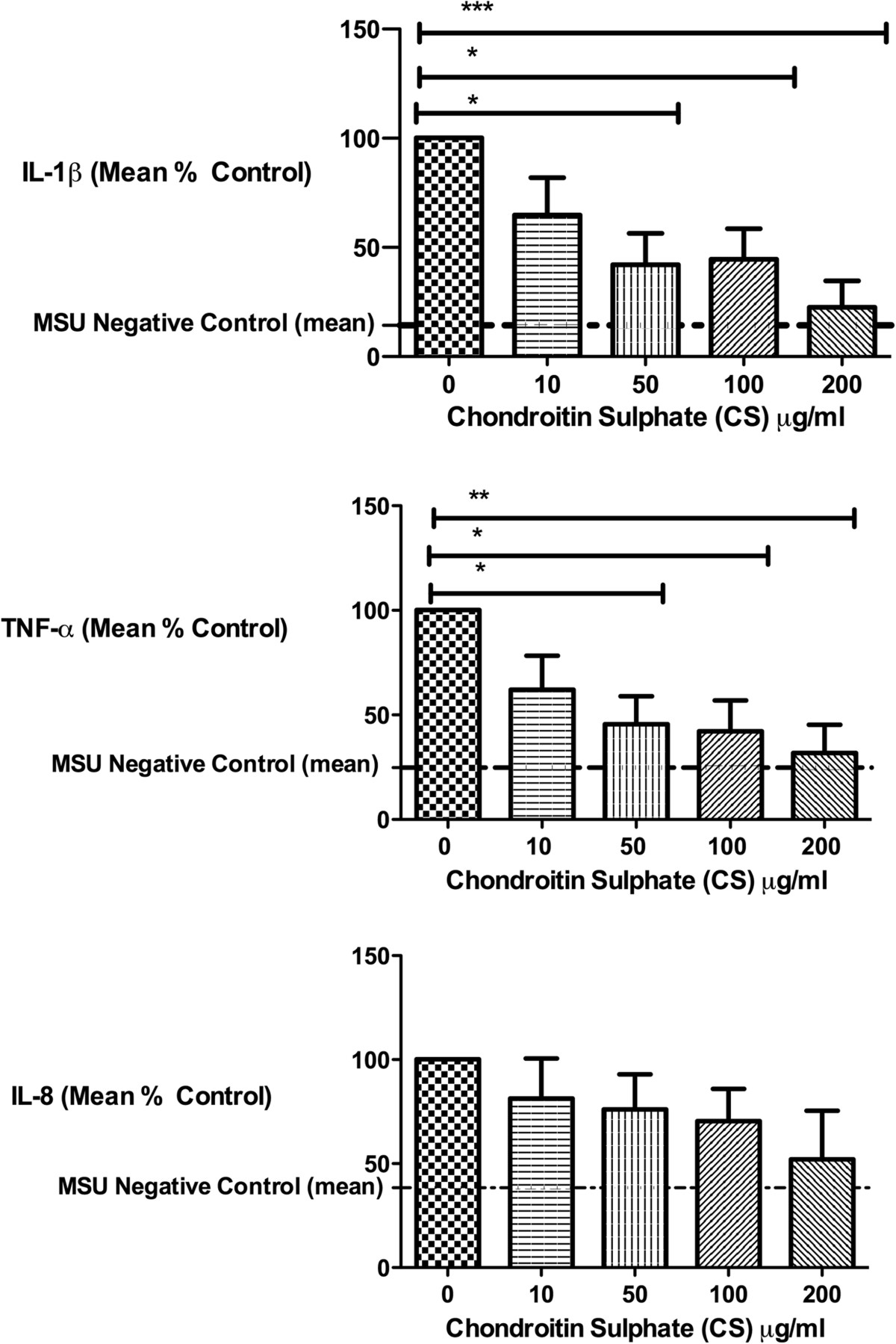
**Chondroitin sulphate (CS) inhibited MSU induced cytokine production.** THP-1 macrophages were pretreated for 4 hours with varying physiological concentrations of chondroitin sulfate (CS, range 0–200 μg/ml) followed by stimulation with MSU (10 mg/dl) for 24 hours. Media were analyzed for cytokines: a) IL-1β, b) TNF-α, and c) IL-8. Production of IL-1β (p = 0.003) and TNF-α (p = 0.02) were significantly inhibited by CS while IL-8 (p = 0.41) showed a similar but non-significant trend. *p ≤ 0.05, **p ≤ 0.01 and ***p ≤ 0.001 generated using One-Way ANOVA with Dunnett’s Post-Hoc Test. Results are expressed as a percent of the positive control (left bar with MSU, no CS). Representative mean (standard deviations) raw cytokine concentrations (in pg/ml) for the MSU stimulated conditions without and with 200 mg/dl CS were as follows: IL-1β 348 (184) and 61 (79); TNF-α 359 (440) and 46 (55); IL-8 8502 (193) and 4428 (4009).

To assess the effect of CS on MSU stimulated cytokine production, we pre-incubated THP-1 macrophages in the absence and presence of CS for 4 hours followed by stimulation with MSU 10 mg/dl. This concentration was chosen as it reliably induced IL-1β production, is representative of hyperuricemia, and is associated with a high incidence of gout[20]. IL-1β, TNFα and IL-8 were induced a mean 7-, 4- and 3-fold respectively by MSU. CS significantly inhibited IL-1β (p = 0.0029) and TNFα (p = 0.0174) production from macrophages in response to MSU (Figure 3). These results were also significant by linear trend analysis (p = 0.001 and p = 0.009 for IL-1β and TNFα respectively). Although IL-8 was similarly inhibited by CS (Figure 3), this trend was not statistically significant by ANOVA (p = 0.4147) but was significant by linear trend analysis (P = 0.05). The linear trend analyses demonstrate a reduction of inflammation by CS in a dose-dependent manner.

Three cytokines associated with gouty inflammation, IL-1β, TNFα and IL-8 were all induced by exposure of activated macrophages to MSU crystals. Macrophage exposure to CS for 4 hours prior to MSU led to a significant dose-dependent decline in production of both IL-1β and TNFα. Many of the anti-inflammatory effects of CS are thought to affect the transcription of various cytokines, such as IL-1β and TNFα. In particular, they are thought to affect various kinases, which in turn block the translocation of NFκB to the nucleus[14]. Others have suggested that NFκB could also affect IL-8 production[21]. Martinon et al. demonstrated that MSU crystals lead to IL-β production through activation of the inflammasome[22]. TNFα is also upregulated by MSU crystals in an experimental animal model of gouty arthritis, but blocking IL-1β (either pharmacologically or genetically) lessened this response[23]. In addition, IL-8, a chemotactic factor responsible for neutrophilic infiltration, was upregulated when MSU crystals were injected in the joints of rabbits; this neutrophil response and the gout related synovitis were attenuated with the use of an anti-IL-8 antibody[24].

Based on the literature, the concentrations of CS used in these experiments are comparable to those suggested to be within a physiologically achievable range[18, 19]. The data presented here suggest there may be a role for CS in preventing flares of gout due to initiation of uric acid lowering agents. To gain potential insights into whether CS might play a role in treatment of active gout flares, future studies are needed to test the effects of CS added coincidentally or after MSU stimulation. Given the low side effect profile of CS, it represents an intriguing treatment option for these scenarios in gout. In particular, CS might synergize with other established treatments for gout thereby making it possible to lower doses or discontinue traditional therapies, particularly in the subset of individuals with relative contraindications to the traditional therapies including allopurinol, NSAIDs and colchicine in the context of renal insufficiency.

A number of meta-analyses have found oral CS to be both safe and well tolerated[12, 13]. However, one must take into account both the purity and source (i.e. bovine or shark etc.) as other *in vitro* studies have shown that *in vitro* anti-inflammatory properties of CS can vary based on the preparation[25–27]. Further studies in humans will be needed to determine if CS has a role as a treatment option for patients with gout.

A limitation of our study was the use of THP-1 cells derived from a human monocytic cell line. They are often employed in the laboratory setting because of their ease of use. However, it would be of benefit to repeat these experiments in primary peripheral monocytes or primary synovial macrophages. Although we established that IL-1β, produced by macrophages in this system, was a product of inflammasome activation, these experiments do not establish the exact target of CS inhibition. CS may be blocking NFκ-B activation, as established by others[18], which would block pro-IL-1β transcript expression. Alternatively, CS could be acting outside of the cell by blocking the interaction of MSU or extracellular matrix fragments with cell surface receptors on macrophages[28]. Still the anti-inflammatory effects of CS and other sulphated glycosaminoglycans may be mediated through sequestering of cytokines as suggested based on NMR and fluorescent spectroscopy as well as computational simulation studies[29]. Finally, CS could have pleiotropic effects on the cell, some of which have yet to be elucidated.

## Conclusions

CS decreased MSU-mediated cytokine production from activated macrophages. In particular, IL-1β and TNFα were lowered in a dose-dependent manner by CS. Given the role of these cytokines in initiating gouty inflammation, CS may have a role as a prophylactic agent in the treatment of gout.

### Ethics statement

This paper represents a series of *in vitro* cell culture experiments using an immortalized cell line (ATCC TIB-202). These studies did not involve any human or animal experiments. Therefore, we did not need the approval of any research ethics committee or institutional review board.

## Authors’ original submitted files for images

Below are the links to the authors’ original submitted files for images. Authors’ original file for figure 1 Authors’ original file for figure 2 Authors’ original file for figure 3

## Abbreviations

ATCC: American type culture collection
CS: Chondroitin sulphate
FBS: Fetal bovine serum
HEPES: 4-(2-hydroxyethyl)-1-piperazineethanesulfonic acid, a commonly used organic ion buffering agent
IL-1β: Interleukin 1β
MSU: Monosodium urate
PBS: Phosphate buffered saline
RPMI: Roswell park memorial institute, medium commonly used in cell culture
TNF: α-tumor necrosis factor α.
