# Supplementary figures and images for: Monosodium urate crystal induced macrophage inflammation is attenuated by chondroitin sulphate: pre-clinical model for gout prophylaxis?
Source: BMC Musculoskelet Disord. 2014 Sep 27;15:318. doi: 10.1186/1471-2474-15-318 (PMC4189145; doi:10.1186/1471-2474-15-318)

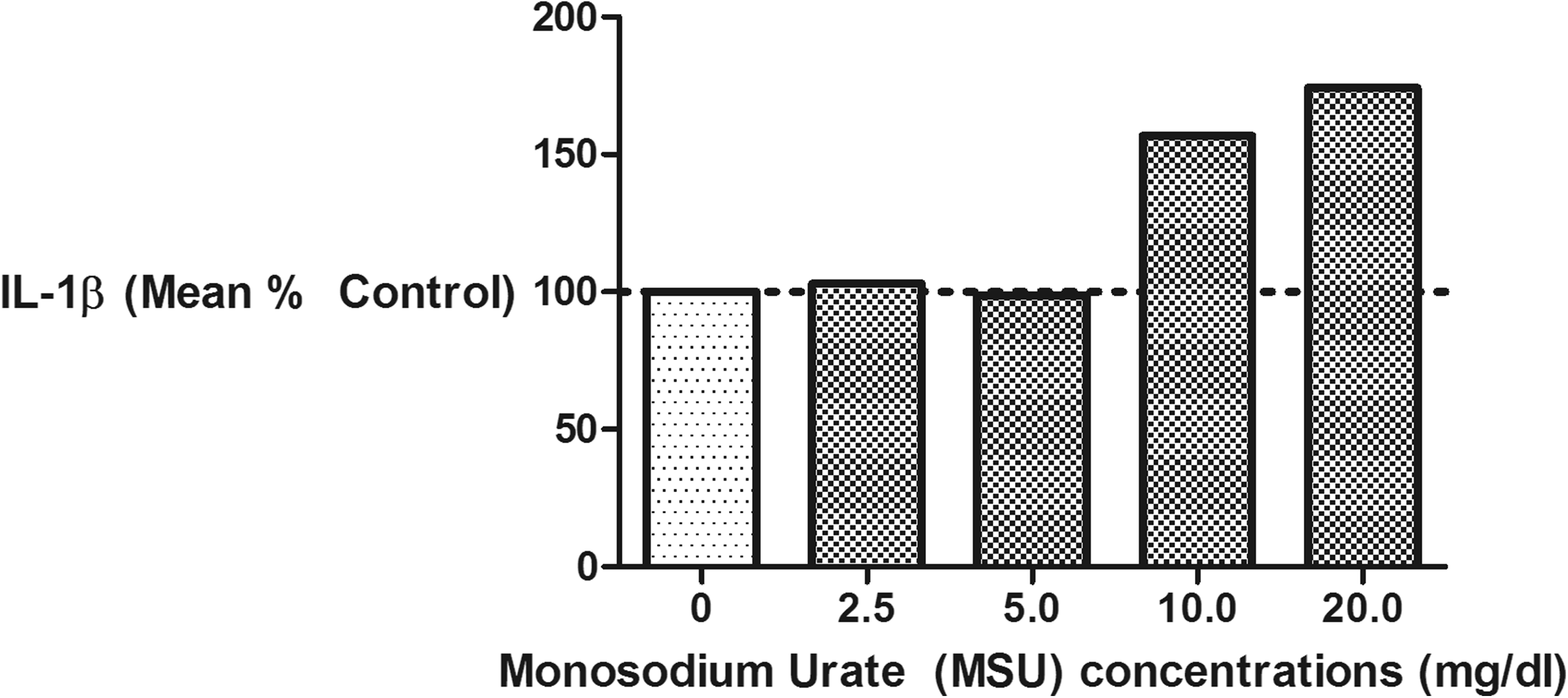

Supplement: Supplementary file 1 — Authors’ original file for figure 1 [file 12891_2014_2252_MOESM1_ESM.tif]

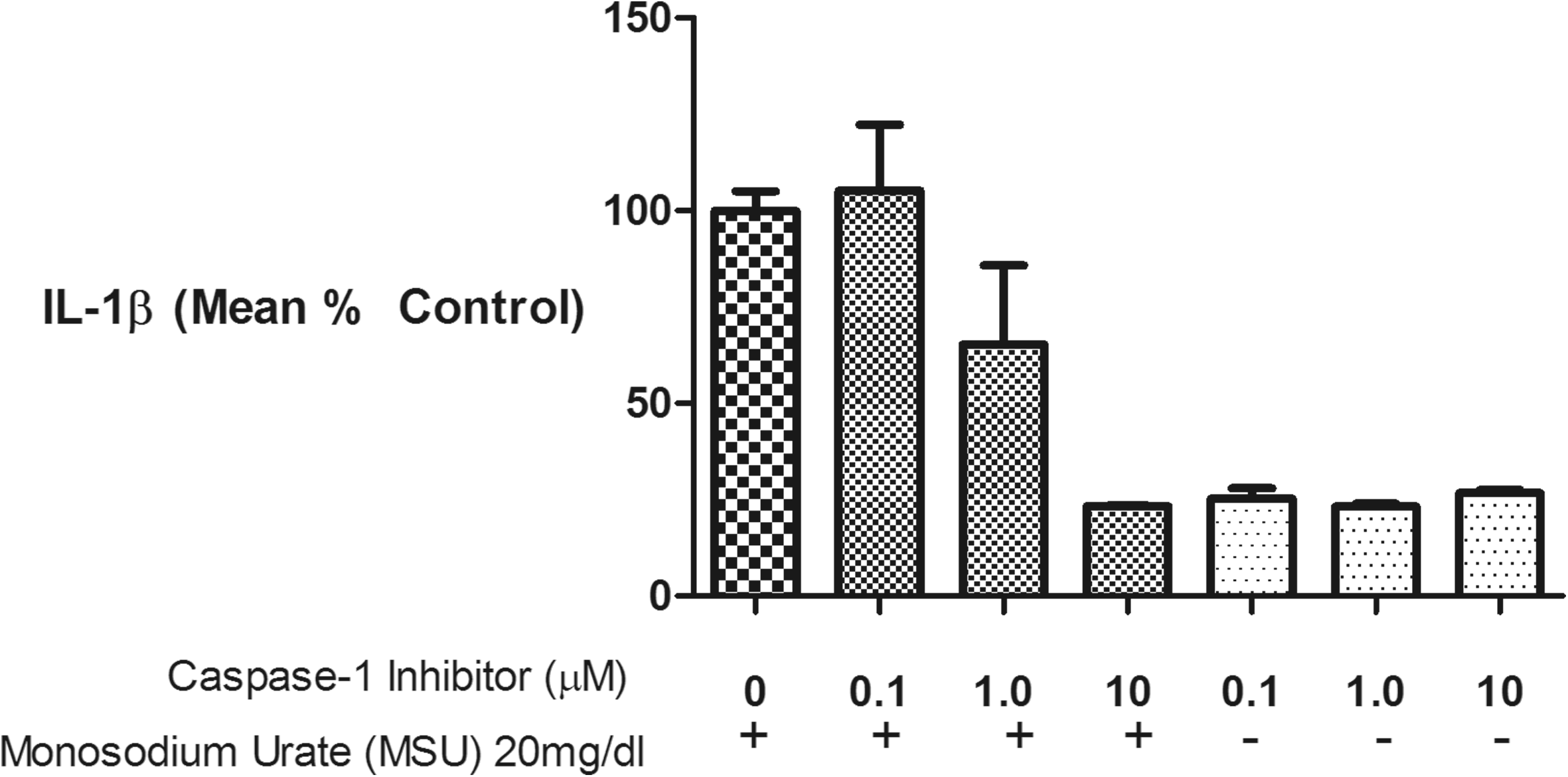

Supplement: Supplementary file 2 — Authors’ original file for figure 2 [file 12891_2014_2252_MOESM2_ESM.tif]

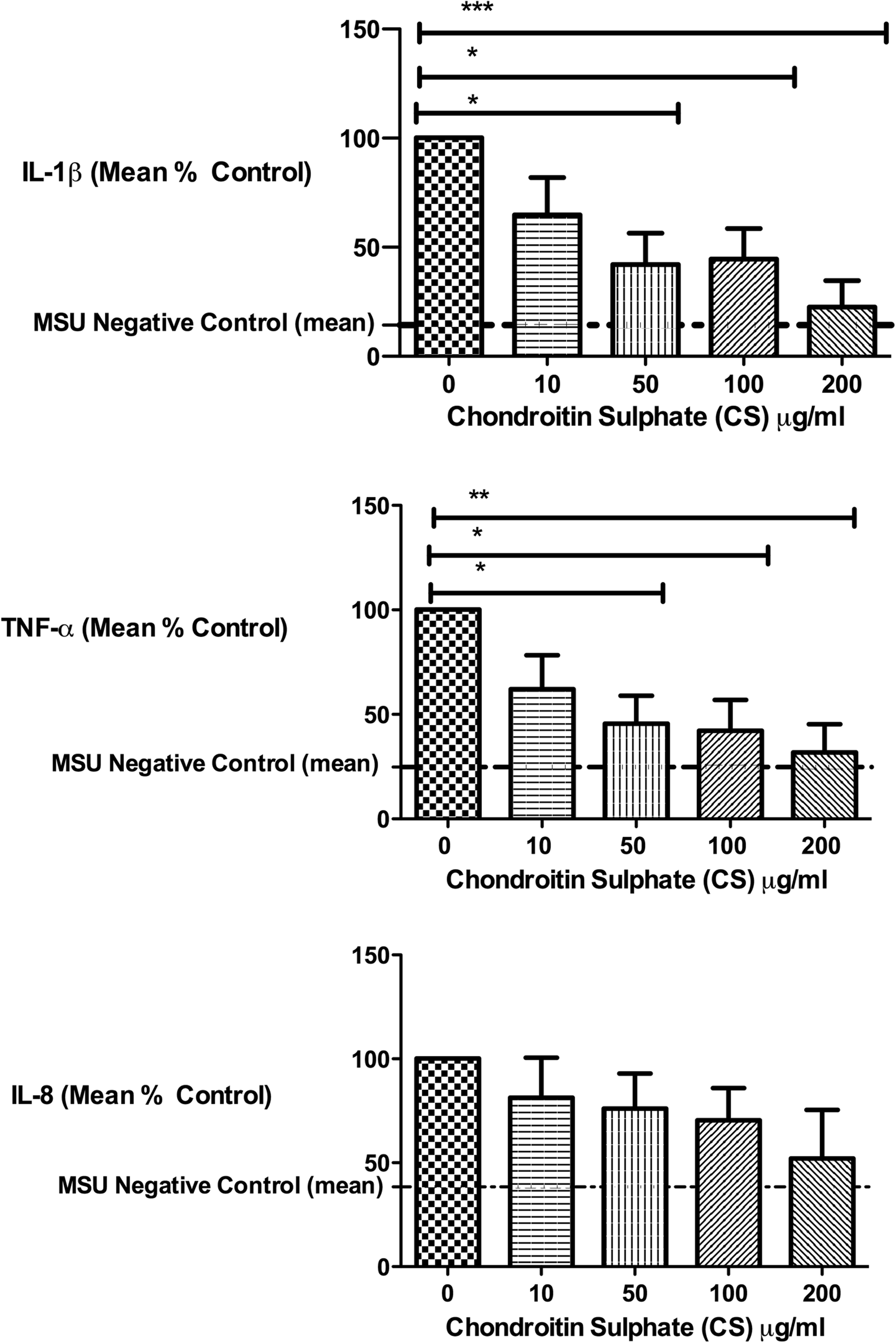

Supplement: Supplementary file 3 — Authors’ original file for figure 3 [file 12891_2014_2252_MOESM3_ESM.tiff]
